# Selecting a Bedside Cognitive Vital Sign to Monitor Cognition in Hospital: Feasibility, Reliability, and Responsiveness of Logical Memory

**DOI:** 10.3390/ijerph16193545

**Published:** 2019-09-22

**Authors:** Padraic Nicholas, Rónán O’Caoimh, Yang Gao, Afsana Habib, Thomas Karol Mross, Roger Clarnette, D. William Molloy

**Affiliations:** 1 Centre for Gerontology and Rehabilitation, University College Cork, St Finbarr’s Hospital, Douglas Road, T12 XH60 Cork, Ireland; 109746021@umail.ucc.ie (P.N.); xhandgy@gmail.com (Y.G.); w.molloy@ucc.ie (D.W.M.); 2 Clinical Sciences Institute, National University of Ireland Galway, Costello Road, H91 TK33 Galway, Ireland; 3 Mercy University Hospital, Grenville Place, T12 WE28 Cork, Ireland; afsana.habib@health.wa.gov.au; 4 Geriatrisches Zentrum Bethanien, Agaplesion Bethanien Krankenhaus, Rohrbacher Str. 149, 69126 Heidelberg, Germany; thomasmross@googlemail.com; 5 School of Medicine and Pharmacology, University of Western Australia, 35 Stirling Hwy, Crawley, WA 6009, Australia; roger.clarnette@health.wa.gov.au

**Keywords:** attention, cognitive screening, cognitive vital sign, logical memory, older adults, delirium, dementia, hospital

## Abstract

Although there is a high prevalence of delirium and cognitive impairment among hospitalised older adults, short, reliable cognitive measures are rarely used to monitor cognition and potentially alert healthcare professionals to early changes that might signal delirium. We evaluated the reliability, responsiveness, and feasibility of logical memory (LM), immediate verbal recall of a short story, compared to brief tests of attention as a bedside “cognitive vital sign” (CVS). Trained nursing staff performed twice-daily cognitive assessments on 84 clinically stable inpatients in two geriatric units over 3–5 consecutive days using LM and short tests of attention and orientation including months of the year backwards. Scores were compared to those of an expert rater. Inter-rater reliability was excellent with correlation coefficients for LM increasing from *r* = 0.87 on day 1 to *r* = 0.97 by day 4 (*p* < 0.0001). A diurnal fluctuation of two points from a total of 30 was deemed acceptable in clinically stable patients. LM scores were statistically similar (*p* = 0.98) with repeated testing (suggesting no learning effect). All nurses reported that LM was feasible to score routinely. LM is a reliable measure of cognition showing diurnal variation but minimal learning effects. Further study is required to define the properties of an ideal CVS test, though LM may satisfy these.

## 1. Introduction

Identifying acute cognitive changes in hospital is challenging [1]. Decline in cognition after admission is multi-factorial and may relate to medical conditions, functional deterioration, pharmacological treatments, or the environment itself [1,2]. Although fluctuating cognition is increasingly considered to be a vital sign, particularly in older adults [3], few short, valid, and reliable cognitive tests are available in hospital to routinely monitor changes in cognitive function [4], where the prevalence of delirium approaches 20% [5] and major neurocognitive disorders such as dementia approach 50% [6,7]. Reflecting this, delirium and dementia are frequently unrecognized in hospitals [2,8]. Although serial assessment is important to monitor cognition in dementia [9], it is challenging as inpatients often experience fluctuations and changes in cognition ranging from normal daily variations to those resulting from delirium [10].

Simple tests, such as the months of the year backwards (MOTYB), focusing on attention may be useful in detecting delirium in hospital [10,11]. Further, short cognitive screening instruments can separate delirium from established cognitive impairment [12]. A brief cognitive or delirium screening instrument could therefore be useful as a “cognitive vital sign” (CVS), which if performed regularly on hospital patients could enable monitoring of cognitive function. Early changes in a CVS, outside the normal or the patients’ previously stable baseline range, might alert staff of an underlying problem or clinical deterioration. An early diagnosis of delirium would prompt the search for a cause of the decline [2], potentially limiting its progression and significant associated costs [13].

Another challenge is how to allocate responsibility for the administration of these instruments in routine clinical practice. While it is suggested that nursing staff may be best placed to monitor a CVS [3], the feasibility of this is unclear. Evidence suggests that healthcare professionals are more receptive to using brief and easy-to-administer tools [14,15]. An ideal CVS should also measure as many cognitive domains as possible and have multiple alternative forms to minimize learning or practice effects [16]. In addition, bias associated with age, education, visual and hearing impairment, manual dexterity, sleep status, and other factors including the time of day may impact upon performance and the reliability of testing and should be considered when selecting a candidate CVS [17,18,19,20].

Logical memory (LM) [21], immediate or delayed verbal recall of a short story, is a test of episodic memory originally included as part of the Wechsler Memory Scale [21]. Logically ordered stories test attention and memory and may be easier to encode for those with pre-existing executive dysfunction [22], suggesting LM may be a useful CVS. LM has been included in formal cognitive screening instruments such as the Quick Mild Cognitive Impairment (Q*mci*) screen [23,24,25,26,27], where its immediate version (recall after only a momentary interval), is the most accurate subtest for identifying early cognitive impairment [28]. It is also used in several longitudinal studies [29,30], though like many tests there are concerns over practice effects [22,31] and test-retest reliability [22] even when using alternative forms [31].

The objective of this study was (1) to measure the reliability and feasibility of a selection of potential CVS candidates including LM, MOTYB, and digit-span testing, (2) to examine if normal diurnal fluctuations in these occur, and (3) to assess if the use of LM as a bedside CVS is feasible in hospital. The study also sought to minimize learning effects by developing new alternative LM stories to identify for that are acceptable to nursing staff.

## 2. Materials and Methods

### 2.1. Patients

Patients admitted to wards in two university teaching hospitals (one acute geriatric hospital and one adult rehabilitation unit) in Cork City, Ireland, in May 2013 were invited to participate. Suitable patients were selected using convenience sampling directed by a neutral party (clinical nurse manager) to minimise selection bias. Patients were assessed for a minimum of three days and up to a maximum of five consecutive days. Patients were included if they were over 18 years of age, clinically stable at baseline (defined as no change in medication dosage or frequency and no recent change or exacerbation in their medical condition on day 1, not receiving end-of-life care confirmed by the clinical nurse manager), if their remaining admission was expected to be greater than three days, and if they were able to provide informed written consent. The study received ethics approval (April 2013) from the Research Ethics Committee of the Cork teaching hospitals (ECM 4 (gg) 07/05/13).

### 2.2. Outcomes

LM is a brief test of verbal recall for a short descriptive story; in this study the immediate version (within 30 s) was used. It has 15 items and is scored on a 30-point scale with two points given for every correct answer [32]. Administration takes less than one minute and alternate validated versions are available [33]. LM evaluates working memory, verbal recall, attention, expressive language, and executive function [21]. It is not biased by age or education [28] and is not affected by visual or physical disability. LM is valid in those with different dementia and mild cognitive impairment subtypes [34].

The other rapidly administered measures of attention scored included digit-span testing (auditory presentation of number lists repeated forwards/backwards), MOTYB, and orientation to time. These tests are also presented in the Appendix A. MOTYB is a well-validated test of attention that when used alone is the most accurate, brief bedside screening test for delirium in hospital [10]. In this study it was scored out of 15 points, one point for each month in order and up to three additional points for time of completion (three points for <20, two for 20–25 and one for >25 s). Five-item digit-span forwards and digit-span backwards were each scored out of 10 points, two for each item recalled in order. Digit-span testing is a useful indicator of delirium and cognitive impairment in hospital [35], with the forwards and backwards versions measuring different aspects of cognition; backwards recall draws on visuospatial processing with both versions assessing attention and working memory [36]. Orientation to time, another useful stand-alone guide to the presence and severity of dementia or delirium in hospitalised patients [37], was scored out of five points (a maximum of five if the stated time was within 30 min, four within 30–60 min, three within 1–2 h, two if within three hours and one point if the patient’s estimate was more than three hours off the actual time). All scores were converted to percentages.

### 2.3. Study Measures and Procedures

A pilot study, sample (*n* = 10), was performed to evaluate the feasibility of using LM and to determine administration time. Participants included in pilot testing were excluded from the final research study. Ten alternate forms of LM, three of which had been validated previously [33], were available for repeat testing (see Appendix A). Slight amendments were made to the layout of the new alternative forms and patient instructions as a result of piloting. Data collection questionnaires were piloted simultaneously to ensure they would be time efficient and accurate in recording the study findings in the test environment. Nurses were trained to administer the tests and asked to complete a questionnaire at the end of the study.

Administration and scoring instructions were developed to standardize the testing. A trial demonstration was performed for nursing staff by the principle investigator (DWM). LM was scored as follows: The patient was first asked to concentrate, and any distractions on the ward, were removed (e.g., radios, televisions, hoovers were switched off). On the first consultation an auditory exam, the whispered voice test [38], was performed to test hearing. Patients were warned they were going to hear a short story and that they should repeat back as much of the story as possible, in any order, immediately after the story ending. Nurses were instructed to read the paragraph at about one second for each word unit until complete and wait for a maximum of 30 s for responses. No hints were provided. The patients were read a different version each time. The other tests were scored in the following fixed order (digit-span forwards, digit-span backwards, MOTYB, and orientation to time), see the attached scoring instructions in the Appendix A.

Administration of LM and the battery of other instruments were performed in random order. Independent assessments were performed twice daily (morning/afternoon) by an expert rater (PN). Each assessor was blind to the other’s results. In addition, prior to scoring the CVS on patients, nurses were asked to document whether they felt the patient’s global cognitive condition was subjectively the same, worse, much worse, better, or much better compared to the previous day, using a simple Likert scale. If patients were deemed to have had a clinical change, further questions were asked to determine the nature of these variations, e.g., were activities of daily living (ADLs) affected? Questions were derived following consultation with dementia specialists (Appendix A).

### 2.4. The Questionnaire

A questionnaire was formulated and administered to nurses to identify their view of the time needed to administer the test, their comprehension of the standardised instructions, and the feasibility of performing the CVS testing routinely on the ward. Each questionnaire was pre-assigned an identification number. It was completed by all 14 nurses who performed CVS testing. The questionnaire consisted of a short demographic page and a number of questions using a “Likert scale” format. Nurses were questioned on time needed to administer, comprehension of the standardised instructions, feasibility to perform on the ward, and their willingness to perform a CVS.

### 2.5. Statistical Analysis

Data were analysed using SPSS version 18.0 (IBM, Armonk, NY, USA). Inter-rater reliability (IRR) between raters (expert rater and nursing staff) and the relationship between LM scores and other tests were evaluated using bivariate Pearson’s correlation analysis; IRR was compared for each patient for each day of the study. Canonical correlation and linear mixed modelling were also used. Daily fluctuations in the score for each test were calculated by establishing each patient’s mean score over the testing period. The largest variation around this mean was then taken (upper limits of normal fluctuation) for each patient and an overall mean variance established for this sample. Variability was explored in a number of ways. Variance components analysis was used to explore total variability of scores and the proportion of that variability attributed to patient factors and day-to-day variability; the proportion explained by the patient is the variability of scores attributed to differences between individuals. The remaining proportion is defined as day-to-day variability, the difference in scores for the same patient between different days. Variation from day to day was also examined using maximum difference from patient-level mean. Learning effects were explored using linear mixed modelling (LMM). This regression technique can be applied where observations are not independent from each other (i.e., observations belonging to the same patient). The study day (number) was used as a predictor of the score as both a categorical and continuous variable with significant results indicating changes in scores over time and suggesting learning effects. Subgroup analysis was performed for age and level of formal education to investigate if greater fluctuations existed in scores within these subgroups.

## 3. Results

### 3.1. Baseline Characteristics

In total, 100 patients were assessed. Sixteen were excluded from the study because they were discharged less than three days after commencing the study (*n* = 12), or their clinical condition worsened (*n* = 4) during the course of their participation. The remaining 84 adult inpatients (47 males/37 females; Ratio 1.3:1), were included: *n* = 38 from a rehabilitation unit and 46 from an acute geriatric ward. All patients were Caucasian. The majority of patients were aged >65 years (*n* = 69, 82.1%). There was an inverse relationship between increasing age and decreasing mean LM scores (Table 1). Thirty patients (37.5%) did not complete second-level education, 42 (50%) completed secondary school only, and 12 (14.3%) completed third-level education. Educational attainment up to secondary level did not reflect mean LM scores in this study population. Those who completed third level had higher mean LM scores (16.17) than those who did not (9.9), *p* < 0.05.

### 3.2. Reliability, Variance Day-to-Day (Diurnal), and Learning Effects

The trained nurses and independent rater examined patients over three to five days. Data sets were analysed and categorised into daily results, (see Table 2). IRR for LM was excellent, increasing from *r* = 0.87 on day 1 (*n* = 41 patients) to *r* = 0.97 by day 4 (*n* = 13 patients), a statistically significant difference, *p* < 0.0001. Linear mixed model regression (*r* = 0.93, *p* < 0.001) and canonical correlation (days 1–3) confirmed overall excellent IRR (*r* = 0.92, *p* < 0.001). Excluding those aged <50 years, did not alter the reliability, with the IRR increasing from *r* = 0.84 to *r* = 0.98 between day 1 and day 4. Using variance components analysis, we found that most variation between scores was due to patient factors; we observed that the largest day-to-day variability was for orientation to time (33.3%) and numbers forwards (17.7%), while the lowest was for MOTYB (4.2%) and LM (9%). We found statistically significant learning effect for numbers forwards and numbers backwards, with scores increasing (improving) significantly in the first three days, indicating learning effects (see Table 2).

Mean LM scores ranged from 0 to 28.7/30 according to age. The mean LM score was 10.7/30. The mean variation in the standard deviation was ± 1.5 and 1.9 points for the trained rater and nurses respectively, indicating that fluctuations of approximately two points from the individual’s baseline were outside normal daily changes in this clinically stable sample (see Table 2). LM scores were statistically the same (*p* = 0.98) from days 1 to 3. A slight rise in mean LM scores was evident on days 4 and 5, but these changes were not statistically significant, though sample numbers were small. After removing the results for younger patients (<50 years), LM scores still showed no change over time, reducing from a mean of 10.4 ± 4.9 on day 1 to 10.3 ± 5.0 points by day 3 (*p* = 0.98).

### 3.3. Comparison of Logical Memory with Short Tests of Attention and Orientation

**Figure 1:**
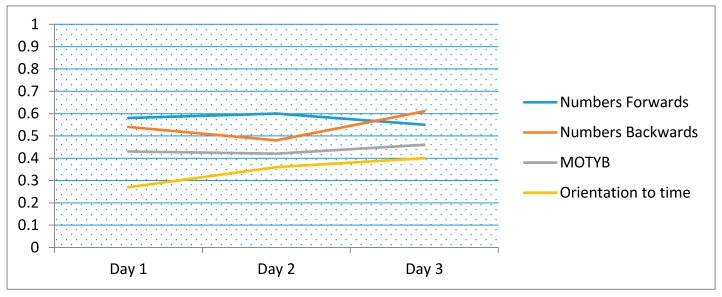
Correlation coefficients for logical memory compared to short tests of attention including months of the year backwards (MOTYB) and orientation (time of the day), over time (days 1 to 3). (Note: 83/84 with complete data included).

**Figure 2:**
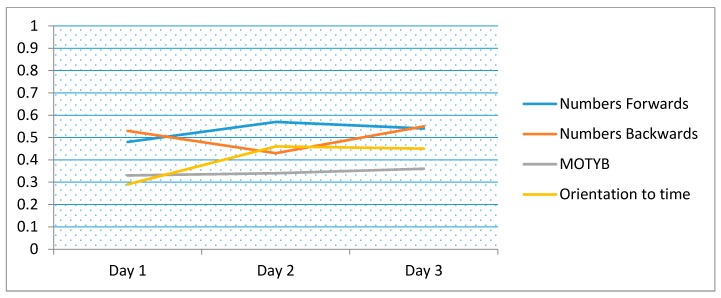
Correlation coefficients for logical memory compared to short tests of attention including months of the year backwards (MOTYB) and orientation (time of the day), over time (days 1 to 3). (Note: *n* = 79, excluding those <50 years).

The correlation between LM and tests of attention (digit-span forwards/backwards, months backwards), working memory (digit-span forwards/backwards) and orientation to time are presented in Table 3 and Figure 1. Data from day 4 and 5 were excluded as sample sizes were considered to be too small. Numbers backwards had the highest consistent correlation with LM (*r* = 0.61). Overall, the correlation between LM and other rapid tests was low. There was an increased correlation between patient orientation to time and LM over time. The correlations were generally lower when younger patients (<50 years old) were excluded, though varied by test. Of note, orientation to time had stronger correlation in older patients >50 years (Figure 2).

### 3.4. Clinical Correlation

Fluctuations in LM scores were then compared to subjective clinical changes in the patients as documented by nursing staff. Nurses observed a global clinical change in 37 patients (44%). In 23 (62%) of these cases, LM score changes reflected nurses’ opinions on the patient’s clinical condition. For the remainder, LM scores did not correlate with their observations. In nine cases, nurses felt the patient’s cognitive condition had become “worse or much worse” as the week progressed. Of these, decreased conversation and social withdrawal were recorded as the primary manifestations (*n* = 6) followed by changes in ADLs (*n* = 2), sleep pattern (*n* = 1), drowsiness (*n* = 1) and anxiety (*n* = 2). Some patients displayed more than one sign of cognitive change.

### 3.5. Feasibility of Using Logical Memory as a Cognitive Vital Sign

A majority of the 14 trained nurses, 86% (*n* = 12), reported that they administered and scored the LM in less than 3 min. Two nurses, aged >60 years, reported taking 3–5 min, although the age of nurse raters and administration time were not statistically significantly associated. All nurses felt the instructions for testers could be understood without difficulty, that LM was a feasible CVS to perform in a busy inpatient environment, and that they would be willing to use it in the future. None of the 14 nurses suggested changes to improve the scoring.

## 4. Discussion

This study presents the reliability, diurnal fluctuations, test, and clinical correlations and perceived feasibility of using LM as a CVS on a daily basis in a stable and predominantly older inpatient population. The results show that LM has high levels of IRR that improved with repeated administration (correlation coefficients range 0.87–0.97). These support other studies showing that the reliability of LM is reasonable [39]. Normal daily fluctuations in LM scores were found, supporting evidence that test-retest reliability is variable [40], though LM scores were statistically the same from days 1 to 3 indicating that unlike other studies [22], there were no statistically significant learning effects evident with repeated LM scoring as the week progressed. The results also show that there was moderate correlation between LM and a battery of short tests of attention, working memory, and orientation (time of the day) that was strongest for numbers forwards and backwards. This is expected as LM tests a wider range of cognitive domains including episodic and working memory [21] compared to simple tests of attention and orientation alone.

Variations in LM scores (fluctuations) correlated with the nurses’ opinion of clinical change in only two-thirds of cases, suggesting that sensitivity was modest. Fluctuations of approximately two out of 30 points were found to be outside of the diurnal variation. However, while fluctuations of this magnitude may be abnormal, meriting further investigation, the likely relatively low sensitivity indicates that changes in LM scores should not be considered in isolation, but viewed in the context of patients’ overall clinical and cognitive status.

The study also compared LM to short tests of attention and orientation often used for detecting delirium and dementia. There was generally only moderate correlation between the LM and these tests, and it was highest for digit-span forwards/backwards. Orientation to time had stronger correlation with LM in older patients, i.e., when those <50 years were excluded. This may be because both orientation [37] and LM [28] are useful tests of cognitive impairment in older patients. Many patients persistently scored towards the upper limits of these other tests, indicating possible ceiling effects. For example, very little variability between individuals was detected for digit-span forwards/backwards suggesting that these tests would not be suitable for a general inpatient population who typically would have a higher cognitive status than the sample tested in this study. This study found that LM and MOTYB had the least tendency towards a learning effect, while digit-span forwards/backwards, possibly due to the repetitive nature of this test, showed evidence of significant learning effects. These highlight potential problems using these other tests in detecting cognitive change when applied on a daily basis, strengthening the case for using LM as a CVS.

Trained nurses perceived that LM was feasible and acceptable to administer as a CVS. The significant increase in IRR as the study progressed may relate to increased assessor and patient familiarity with the test, providing more stable results over time. This suggests that it is beneficial to perform an initial recording (trial run), to improve patient concentration and understanding of the screen, before taking a baseline score. The small variation between the expert and nurse raters shows that scoring LM is not dependent on experience once the standardised instructions are followed. As several nurses scored the same patients (attending nurses changed between shifts and sections), the findings also indicate that LM, when used as a CVS, can be scored by multiple raters, enabling continuity of cognitive monitoring. The CVS was administered in less than three minutes by 86% of nurses. Few brief tests are as time efficient in what is a recognised time efficacy trade-off [41].

### Strengths and Limitations

Strengths include that all cognitive tests were performed concurrently and that training and standardisation were conducted thoroughly before testing. There are a number of limitations. The sample included in this study was small (no sample size calculation was performed) and selected by convenience, which may have introduced bias. However, this was a feasibility study and patient sampling in this way was required to ensure that inappropriate patients, e.g., those with severe illness, active delirium, or those receiving end-of-life care at baseline, were not included. While this limits the study findings to those who were stable, it was required to measure normal day-to-day (diurnal) variability in a steady state and show the potential of these instruments to reliable. Further study to correlate change outside of this normal variability with the onset of delirium is now required. Similarly, no gold standard measure of delirium or cognition was used in this study, meaning it was not possible to correlate or associate scores with confirmed diagnostic states. Baseline cognition was not assessed, and it is possible that some variation in scores may have related to pre-existing cognitive disorders, e.g., there was some indication that a “sundowning” effect may have influenced evening testing, but it was not possible to confirm. No follow-up of patients was conducted, which also reduces the ability to interpret the findings. Likewise, no independent measure of change was recorded. Instead, LM was correlated with established bedside tests of attention and orientation such as MOTYB [10] and staff nurses’ global subjective opinion of change, recorded prior to each assessment. Although this may have created bias, nurses were instructed to record this before and not after administering the CVS. The repetitive nature of many brief bedside cognitive or delirium tests is a fundamental weakness in conducting daily testing [42,43] and monitoring for cognitive change. It facilitates learning effects with repeated use. To minimise this, we used multiple formats for LM (10 variations) and ensured patients were given different forms for each administration. However, only three had previously been validated, potentially resulting in bias. This said, all 10 variations had the same identical structure only varying slightly and were developed by the designers of the Q*mci* screen of which LM is a subset. All participants in this study were Irish nationals of Caucasian ethnicity; this limits external validity and generalisability. There were also potential environmental influences, which may have affected testing and created bias; patients may have scored better for example (e.g., in orientation) if they were rehabilitating rather than in an acute ward or had been listening to the news, had breakfast, or recently checked the time in anticipation of cognitive testing or attending scheduled therapy appointments. This however, reflects real-life practice, arguably a strength of the study.

## 5. Conclusions

In summary, LM appears to be a reliable and efficient candidate for a CVS for use in routine ward practice, a brief test that could detect changes in cognition while in hospital to indicate delirium and those who require specialist referral and assessment. It was also deemed acceptable, easy, and quick to administer by nurses working in clinical practice. It showed moderate correlation with established tests of attention, working memory, and orientation. However, this study was not designed to show superiority of one instrument over another or predictive accuracy for delirium or other acute mental status changes. Instead, this pilot study focused on the reliability and feasibility of LM. Further research is needed to compare it with other short delirium and cognitive screens such as the 4AT and 6-Item Cognitive Impairment Test [12] as well as established standards for diagnosing cognitive impairment and delirium, in a less select sample of consecutive admissions with and without established cognitive impairment and to investigate whether incorporating it into assessments such as the modified early warning score [44] could promote time-efficient detection of early cognitive changes, leading to better use of resources, rapid treatment if required, and improved patient outcomes.

## Figures and Tables

**Table 1:** Age make-up of the cohort * and their associated mean logical memory (LM) scores.

Patients (%)	84	5 (6%)	10 (11.9%)	31 (36.9%)	33 (39.2%)	5 (6%)
Age	Total	18–49	50–65	66–79	80–89	≥90
Mean LM score	10.7	17.6	10.2	11.81	9.7	7.20

* Expressed as a percentage of the sample group in question.

**Table 2:** Variation analysis with mean ± standard deviation (SD) scores for each test over time including logical memory (LM), numbers forwards and backwards, months of the year backwards (MOTYB), and orientation to time.

Test	LM (Independent Rater)	LM (Trained Nurses)	Numbers Forwards	Numbers Backwards	MOTYB	Orientation (Time)
**Overall scores**						
Mean ± SD	10.9 ± 5.5	10.9 ± 5.9	9.1 ± 1.4	5.1 ± 2.4	11.1 ± 4.1	4.2 ± 1.0
**Scores by day of administration (Mean ± SD)**						
Day 1	10.9 ± 5.4	10.9 ± 5.4	8.8 ± 1.6	4.8 ± 2.6	11.1 ± 4.1	4.2 ± 1.2
Day 2	10.7 ± 5.1	11.5 ± 5.7	9.3 ± 1.3	5.3 ± 2.4	11.1 ± 4.1	4.2 ± 0.9
Day 3	10.8 ± 5.5	10.5 ± 5.9	9.3 ± 1.4	5.3 ± 2.3	11.3 ± 4.1	4.0 ± 0.9
Day 4	11.5 ± 7.3	11.5 ± 8.0	9.7 ± 0.8	4.3 ± 2.2	10.3 ± 4.0	4.3 ± 0.7
Day 5	12.3 ± 5.9	9.3 ± 5.9	10.0 ± 0.0	5.3 ± 1.2	11.7 ± 3.5	4.7 ± 0.6
**Variance components analysis (% of variance explained by various factors)**						
Time (day of follow-up)	0.3%	0.8%	3.5%	1.5%	0.3%	1.0%
Patient	90.7%	86.7%	78.7%	88.7%	95.5%	65.7%
Day-to-day variability	9.0%	12.5%	17.8%	9.8%	4.2%	33.3%
**Variation from day to day based on max difference from patient-level mean**						
Mean/Median	1.8/1.3	2.2/2.0	0.5/0.0	1.0/1.3	0.9/0.7	0.7/0.7
% overall mean	16.6%	20.3%	5.9%	18.8%	7.7%	17.2%
% patients with zero fluctuation	11.9%	14.6%	63.1%	28.9%	32.1%	10.8%
**Variation from day to day (based on standard deviation at the patient level)**						
Mean/Median	1.5/1.2	1.9/2.0	0.5/0.0	0.8/1.2	0.8/0.6	0.6/0.6
**Learning effect (Linear Mixed Model, significance of study day)**						
Categorical	*p* = 0.70	*p* = 0.54	*p* < 0.001 ^a^	*p* = 0.001 ^c^	*p* = 0.23	*p* = 0.28
Continuous	*p* = 0.30	*p* = 0.33	*p* < 0.001 ^b^	*p* = 0.002 ^d^	*p* = 0.40	*p* = 0.46

^a^ There is a significant difference between day 1 and day 2 (*p* < 0.001) and between day 1 and day 3, *p* < 0.001; ^b^ Numbers forward scores increase significantly by an average of 0.21 points/day (95% CI 0.11–0.31), *p* < 0.001; ^c^ There is significant difference between day 1 and day 2 (*p* = 0.003) and between day 1 and day 3, *p* = 0.003; ^d^ Numbers backwards scores increase significantly by an average of 0.20 points/day (95% CI 0.07–0.33), *p* = 0.002.

**Table 3:** Correlation coefficients (Spearman’s rho) for logical memory and short tests of attention including months of the year backwards (MOTYB). (Note: *n* = 83/84 with complete data included; values day 4 and 5 were excluded).

Days	Digit-Span Forwards	Digit-Span Backwards	MOTYB	Orientation to Time
Day 1	0.58	0.54	0.43	0.27
Day 2	0.60	0.48	0.42	0.36
Day 3	0.55	0.61	0.46	0.40

**Figure A1:**
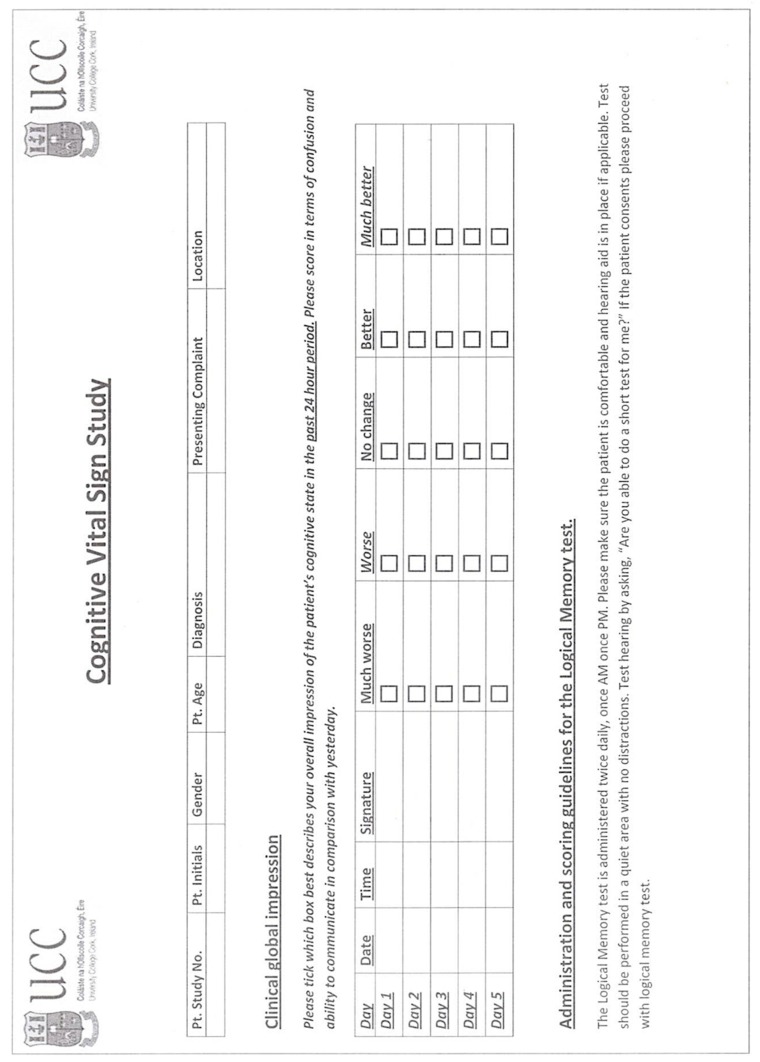

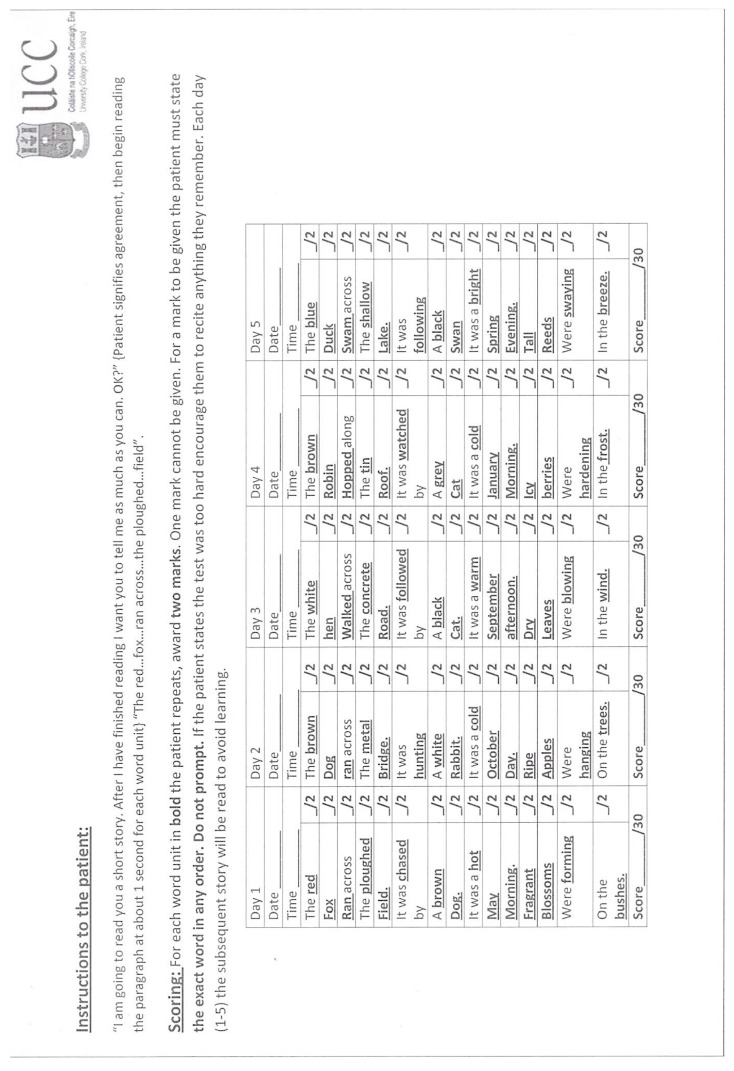

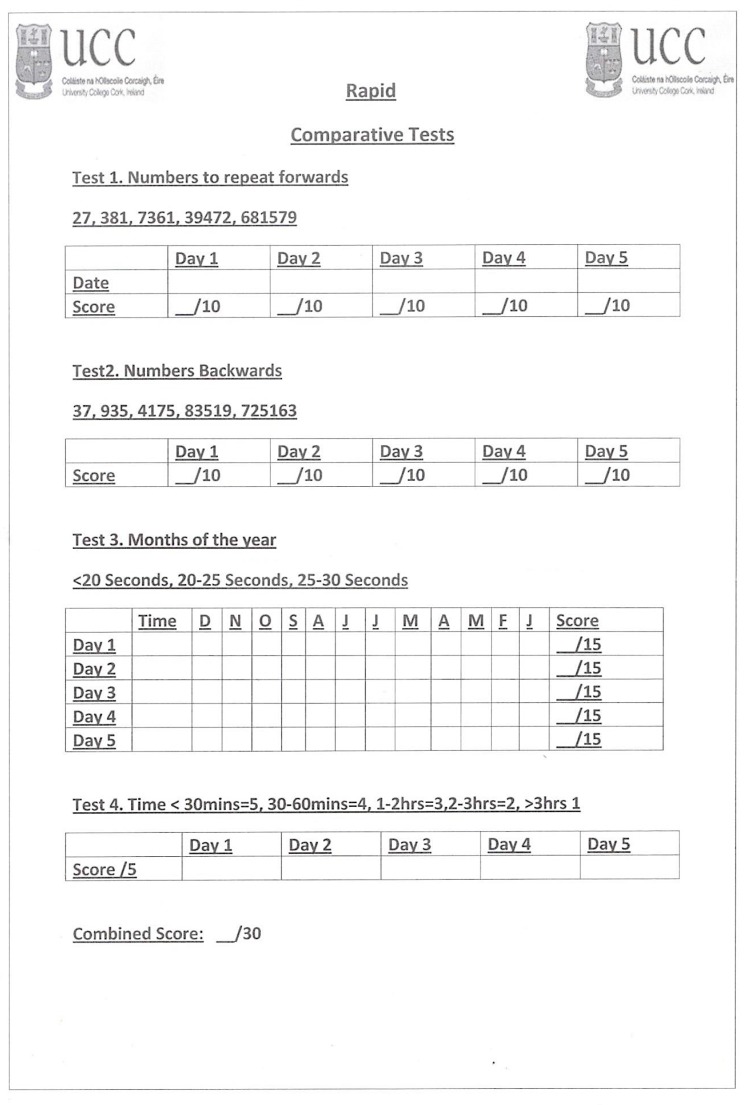
Outcome measures including logical memory test score sheets with alternative versions.

**Figure A2:**
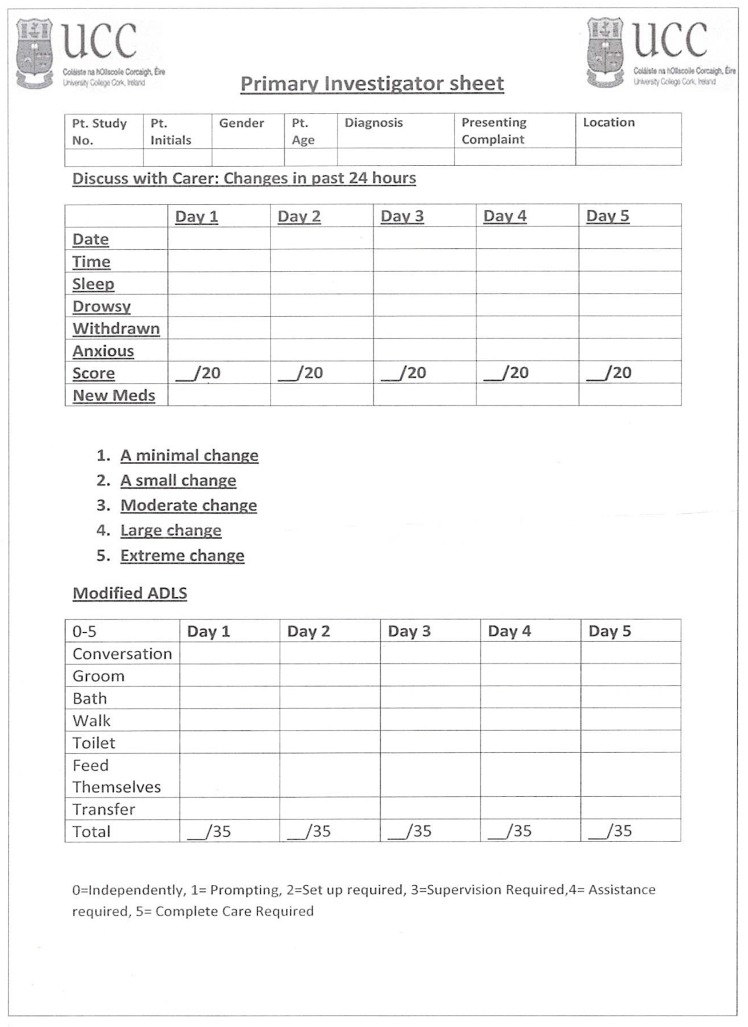
Investigator score sheets documenting clinical status.

**Figure A3:**
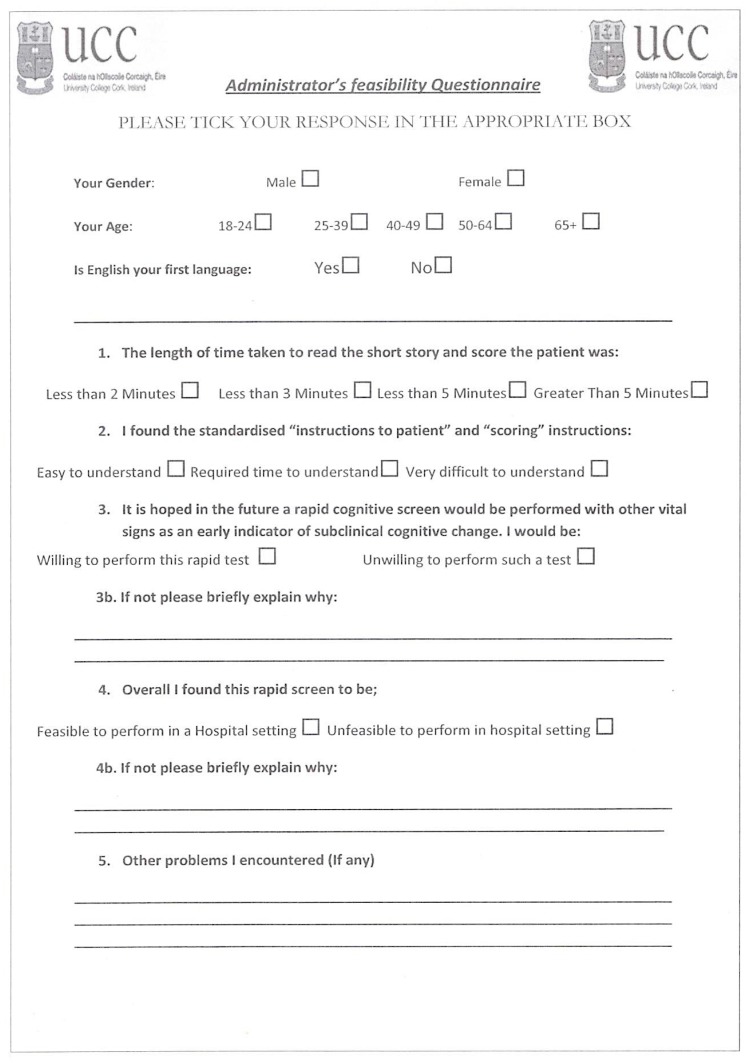
Feasibility questionnaire.
